# Surgical management of lung, liver and brain metastases from gynecological cancers: a literature review

**DOI:** 10.1186/s40661-016-0028-3

**Published:** 2016-06-17

**Authors:** Neville F. Hacker, Archana Rao

**Affiliations:** Gynaecological Cancer Centre, Royal Hospital for Women, Randwick, NSW 2031 Australia; School of Women’s and Children’s Health, University of New South Wales, Kensington, NSW 2052 Australia

**Keywords:** Gynecological malignancy, Cervix, Endometrium, Ovary, Metastasis, Brain, Lung, Liver, Survival

## Abstract

**Background:**

The management of patients with recurrent gynecological malignancy is complex, and often contentious. While historically, patients with metastases in the lungs, liver or brain have been treated with palliative intent, surgery is proving to have an increasing role in the management of such patients.

**Methods:**

In this review article, the surgical management of lung, liver and brain metastases from gynecological cancers is examined. A search of the English language literature over the last 25 years was conducted using the Medline and PubMed databases.

**Results:**

The results for management of metastases from the endometrium, ovary and cervix to the lung, brain and liver show that surprisingly good long-term survival results can be achieved for resection of metastases from all three organs. Patient selection is critical, and surgery is often used in conjunction with other treatment modalities.

**Conclusions:**

From this review, it is apparent that surgery should play an increasing role in the management of patients with parenchymal metastases from gynecological cancers. The surgery should ideally be performed in high volume, tertiary centers where there is a committed multi-disciplinary team with the necessary infrastructure to achieve the best possible outcomes in terms of both survival and morbidity.

## Background

The primary management of patients with a gynecological malignancy is usually protocol driven, and is seldom controversial, but the management of patients with recurrent disease is often contentious. It requires a multidisciplinary team discussion, and complex decisions around the possible roles of surgery, chemotherapy, radiation therapy or hormonal therapy. Often, a combination of therapies will be required.

Historically, patients with metastases in the lungs, liver or brain have been treated with palliative intent. Surgery is proving to have an increasing role in the management of such patients, and survivals are surprisingly good in many cases.

We undertook a search of the English literature over the past 25 years to seek references to the surgical management of lung, liver or brain metastases from cancers of the endometrium, ovary or cervix. The MEDLINE/PubMed database was searched, using the keywords metastases, lungs, liver, brain, endometrium, ovary and cervix.

## Surgical management of lung metastases

The first successful lobectomy for lung metastases in the 20^th^ century was reported by Barney and Churchill in 1939 [1]. The patient was a 55-year old woman with a metastatic renal cell carcinoma, and she survived disease-free for 23 years Subsequently, there have been a number of reports of pulmonary metastasectomy for a variety of tumors [2–8], and the commonest primary epithelial tumour sites have been the colon, rectum, kidney and breast [9].

Endometrial cancer is the commonest gynecological cancer in Western countries, and the majority of patients are diagnosed with disease confined to the corpus [10]. The lungs are the commonest site of hematogenous spread for patients with advanced endometrial cancer [11, 12], but lung metastasis may occasionally occur with very early stage disease [13].

The commonest gynaecological cancer in developing countries is cervical cancer [14]. A review of the records of 2075 Sri Lankan women treated for cervical cancer from 1989 to 1993 reported that 38 patients (1.8 %) developed lung metastases, with a median interval from diagnosis of 9 months [15].

### Diagnosis

Most recurrences in the lungs are diagnosed by the investigation of symptoms, or of rising CA125 titers in the case of ovarian cancer. Barter et al. reported that there was no indication for routine chest x-ray in the follow-up of patients with cervical cancer, as there was no significant survival difference between symptomatic and asymptomatic patients [16].

### Imaging

Thoracic metastases from gynecologic malignancies exhibit various imaging patterns [17]. Metastases from endometrial cancer typically manifest as pulmonary nodules and lymphadenopathy, whereas ovarian cancer often manifests with small pleural effusions and subtle pleural nodules. Most squamous cervical carcinomas manifest as solid pulmonary nodules, but cavitation occurs reasonably frequently. A “halo sign” is sometimes seen in hemorrhagic metastatic choriocarcinoma. Metastases from common gynecologic malignancies may be subtle and mimic benign condition such as intrapulmonary lymph nodes or granulomatous disease [17]. If a solitary lung lesion is found, it is always important to consider the possibility of primary lung cancer.

The spiral computed tomographic (CT) scan has revolutionized the identification of small lung metastases. Before the advent of spiral CT technology, bimanual palpation of the lung through an open thoracotomy or sternotomy was considered necessary to avoid missing small lesions [9]. The spiral CT scan has allowed better characterisation of both location and resectability of pulmonary nodules, so minimally invasive surgery has become a more attractive option [18].

A preoperative PET/CT is desirable to exclude disease beyond the lungs, which would make pulmonary resection inadvisable in most cases.

### Indications for pulmonary metastasectomy

Specific criteria vary considerably in the literature. Clearly, there needs to be an adequate pulmonary reserve, and a limited number of lung metastases. A solitary metastasis is ideal, and 2 papers have reported a 100 % 5-year survival for a total of 21 patients with a solitary endometrial lung metastasis treated with wedge resection and adjuvant hormonal therapy [11, 19]. The estrogen and progesterone receptor (ER/PR) status should be obtained at the time of metastacectomy for endometrial cancer [20].

Except for patients with ovarian cancer, whose tumors are often quite sensitive to chemotherapy or targeted therapy, it is preferable to have no spread beyond the thorax. A disease-free interval of at least 12, but preferably 24 months, is an important prognostic factor [9, 19, 21–23].

### Surgical technique

The operative procedure of choice is a wedge resection. Seki et al. stated that this should be performed with a disease-free margin of at least 2 cm for patients with metastatic lesions smaller than 3 cm diameter. Lobectomy was recommended for lesions larger than 3 cm, because of the greater risk of microscopic satellite lesions [24].

Although most reports are of open thoracotomies, video-assisted thoracic surgery (VATS) has recently become an accepted and often preferred modality in patients with a limited number of metastases, either unilateral or bilateral [18]. It is usually associated with a shorter hospital stay, and preserves the ability of the patient to undergo repeated resections, which may be necessary to achieve cure [9].

Mediastinal lymphadenectomy was recommended by Seki et al. for metastatic squamous cell carcinomas 3 cm or more in diameter [24]. In the paper from the Mayo clinic, there was no association between lymphadenectomy and survival, but the number of positive nodes was small [23]. On the evidence available, resection of at least bulky nodes only would seem to be a reasonable option.

### Reported series

The findings of reported series with 5 or more patients are summarised in Table 1. Although small series had been reported earlier [2, 25, 26], the first major report of resection of pulmonary metastases from a gynecological cancer came from Memorial Sloan Kettering Cancer Center in 1992 [27]. The study involved 45 patients whose pulmonary metastases from uterine sarcomas were resected between 1960 and 1989. All patients had a prior hysterectomy for uterine sarcoma, no extrathoracic tumor, and disease that was thought to be resectable. The mean age of the patients was 50 years, and the mean interval from hysterectomy to thoracotomy was 44 months (range 1 to 193 months).

**Table 1:** Major series reporting surgical management of pulmonary metastases from gynaecological malignancies

First Author Year of Publication	Number of Cases	Primary Tumour	Pattern of Metastasis	Surgery	Survival/Recurrence Outcomes	Prognostic Factors
Adachi [29] 2015	23	Epithelial gynecologic cancers Major series reporting suCervical – 60.9 % Endometrial – 17.4 % Ovarian – 21.7 %	1 nodule – 69.6 % 2–3 nodules – 30.4 %	VATS – 56.5 % Conventional thoracotomy – 43.5 %	5 year OS: Cervical – 61 % Endometrial – 100 % Ovarian – 100 % Recurrence – 43.4 %	Univariate analysis – positive prognostic factors for survival: Endometrioid vs mucinous adenoca DFI >2 years
Gonzalez Casaurran [19] 2011	27	Uterine and cervical cancer	1 metastasis – 66.7 % 2 metastases – 18.5 % ≥2 metastases – 14.8 %	Surgical approach: - thoracotomy – 81.5 % - sequential bilateral – 7.4 % - unilateral VATS – 3.7 % - sequential bilateral VATS – 3.7 % - VATS + thoracotomy – 3.7 % Lung resection: - Wedge resection – 81.5 % - Lobectomy – 11.1 % - Other – 7.4 % Second surgery for metastases - 18.5 %	Median survival from diagnosis of metastases – 94 months 5-year OS after diagnosis of metastases – 84.1 % Overall relapse rate 44 %	Positive prognostic factors Primary site – endometrial vs cervical (*P* = 0.023) DFI >24 months (*p* = 0.054)
Burt [22] 2011	82	Sarcoma Included male and female patients Leiomyosarcoma – 31 cases (38 %) - 24 pts (77 %) of these were female In female pts, uterus most common primary site	Solitary metastases – 16 pts (52 %) Bilateral disease – 19 %	- Wedge resection – 71 % - Lobectomy – 23 % - Segmentectomy – 6 % - VATS – 58 %	5 year survival: - Leiomyosarcoma – 52 % - other sarcoma – 32 % 2^nd^ pulmonary metastasectomy – 58.5 % Second pulmonary recurrence - 30.5 %	Multivariate analysis – DFI >12 months from time of primary tumour resection
Lim [90] 2010	21	Primary and recurrent cervical cancer	Not reported	23 resections in 21 patients - thorocatomies – 43.5 % - VATS – 52.2 % - VATS following thoracotomies – 4.3 % Procedures – 49 - wedge resections – 51.1 % - lobectomies – 18.4 % - mediastinal LN dissections – 24.5 % - segmentectomy – 2.0 % - diaphragmatic resection −2.0 % - pleurectomy – 2.0 %	Note – only 14 patients had recurrent cervical cancer, and 1 patient had primary lung cancer and mediastinal LN metastasis from cervical cancer Median f/up 16 months (range 2–67) - 2 pts died of disease - 3 pts alive with disease - 16 pts alive without disease	Not reported
Clavero [23] 2006	70	Uterine corpus – 52.9 % Endometrium – 32.9 % Cervix – 10.0 % Ovaries – 2.9 % Vagina – 1.3 % Histopathology: Leiomyosarcoma – 41.4 % Adenocarcinoma – 32.9 % Other sarcoma – 15.7 % SCC −7.1 % Other – 2.9 %	Median number of lung metastases – 2 (range 1–19)	Wedge excision – 63 % Lobectomy – 20 % Bilobectomy – 3 % Pneumonectomy – 2.5 % Combination – 12.5 %	5-year OS 46.8 % (95 % CI 34.2-63.0 %) 10 year OS – 34.3 % (95 % CI 19.7-52.5 %)	Factors that adversely affected survival: DFI between 1^st^ gynecologic procedure and pulmonary resection <24 months (*p* = 0.004) Primary site in cervix (*p* < 0.001)
Yamomoto [28] 2004	29 (out of 7748 = 0.37 %)	Cervical cancer (Stage Ib or II treated with curative intent surgery or radiotherapy)	Solitary metastasis – 58.6 % Multiple metastases – 43.4 %	Wedge resection – 27.6 % Segmentectomy – 6.9 % Lobectomy – 65.5 % Hilar or mediastinal lymph node dissection – 55.2 %	5 year DFS after pulmonary metastasectomy – 32.9 %	For DFS: - ≤2 metastases - SCC
Anraku [21] 2004	133	Uterine malignancies (cervix and endometrium) Histopathology: SCC – 43.6 % Cervical adenocarcinoma – 9.8 % Endometrial adenocarcinoma – 17.3 % Choriocarcinoma – 12.0 % Leiomyosarcoma – 4.7 %	Solitary metastasis −58 % 2–3 mets – 23 % ≥4 mets – 17 %	Wedge resection – 50 % Lobectomy – 45 % Bilobectomy – 2.5 % Pneumonectomy – 2.5 %	Overall survival after surgical resection: 5-year – 54.6 % 10-year – 44.9 % 5-year survival by histpathological type: SCC – 46.8 % Cervical adenoca – 40.3 % Endometrial adenoca – 75.7 % Choriocarcinoma – 86.5 % Leiomyosarcoma – 37.9 %	Univariate analysis – negative prognostic factors: Primary tumour in cervix DFI <12 months Resection ≥ 4 mets Large tumour size (≥3 cm) Multivariate analysis: DFI < 12 months
Anderson [20] 2001	82 eligible pts 25 underwent pulmonary resection	Eligible patients: Uterine – 73.2 % Cervical – 26.8 % Patients undergoing resection: Uterine – 76.0 % Cervical – 24.0 %	Solitary – 28 % Multiple – 72 %	Uterine: - Wedge – 63.1 % - Lobectomy – 10.5 % - Lobectomy/wedge – 15.8 % - Bilobectomy – 5.3 % - Segmentectomy – 5.3 % Cervix: - Wedge – 66.7 % - Lobectomy – 33.3 %	Uterine cancer - median survival 26 months - Leiomyosarcoma −25 months - Adenocarcinoma – 46 months Cervix cancer - median survival 36 months	Uterine cancer – favourable prognostic factors: Leiomyosarcoma vs adenocarcinoma (*p* = 0.02)
Levenback [27] 1992	45	Uterine sarcomas: - Leiomyosarcoma – 84 % - Endometrial stromal sarcoma – 9 % - Mesodermal mixed tumours – 7 %	Unilateral lesions – 71 % 1 lesion – 51 % Nodules >2 cm – 70 %	Staged thoracotomies – 100 % Median sternotomy and bilateral resections – 4.4 % Incomplete resection – 36 %	From time of pulmonary resection: - 5 year survival – 43 % - 10 year survival – 35 % Median follow-up – 89 months Disease recurrence – 42 %	Significant predictors: - unilateral vs bilateral disease Not significant: - metastasis size - number of metastases - disease free interval - patient age

*VATS* video-assisted thoracoscopic surgery, *OS* overall survival, *DFI* disease free interval

All gross disease could be resected in 29 patients (64 %), the vast majority by wedge resection. The postoperative mortality was 2 % (one patient). The 5- and 10-year survival from the time of the pulmonary resection was 43 % and 35 % respectively, with a mean follow-up of 25 months. The mean survival for patients with bilateral disease was 27 months, while it was 39 months for patients with unilateral disease (*p* = 0.02).

A more recent paper looked at 82 male and female patients who underwent pulmonary resection for metastatic sarcoma with curative intent at the Brigham and Women’s Hospital from 1989 to 2004 [22]. Leiomyosarcomas accounted for 31 cases (38 %), and 77 % of leiomyosarcomas were in females. Patients with leiomyosarcomas had a better overall survival than patients with other sarcoma subtypes (70 versus 24 months; *p* = 0.049). Disease-free survival of greater than 12 months from time of primary tumor resection was the only significant prognostic factor in multivariate analysis. Systemic chemotherapy had no significant effect on long term survival.

In 2004, the Metastatic Lung Tumor Study Group of Japan reported the results of the largest series of patients having pulmonary metastasectomy for uterine malignancies. They reported on 133 patients undergoing surgery between March 1984 and February 2002 [21]. The postoperative mortality was 0.8 % (1 case). The only significant prognostic factor in multivariate analysis was a disease-free interval of less than 12 months [21].

Another Japanese study published in 2004 evaluated the role of resection of pulmonary metastases from patients with stage Ib or II cervical cancer who underwent curative initial treatment [28]. The 5-year disease-free survival was 42 % for patients with one or two metastases, compared to 0 % for patients with three or four (*p* = 0.0003). Patients with squamous cancers had a 5-year disease-free survival of 47.4 %, compared to 0 % for patients with glandular cancers (*p* = 0.014)

A large series of surgical resections for lung metastases from gynaecological malignancies was reported from the Mayo Clinic in 2006 [23]. They reported 70 patients with metastatic disease limited to the lungs who were treated between 1985 and 2001. Synchronous lung metastases were present in 9 patients (13 %). Post-operative morbidity occurred in 18 patients (26 %), and there was one postoperative death (1.4 %) [23]. The overall survival for the group was 47 % at 5 years and 34 % at 10 years.

The most recent report was from Nagoya University Hospital in 2015 [29]. They reviewed 37 patients with isolated lung metastases (<3 nodules). They compared 23 patients who underwent surgical resection (cervical (14), endometrial (4) or ovarian (5) carcinomas), with 10 patients who underwent chemotherapy only. Among 6 patients who recurred in the lung a second time, 5 underwent a second pulmonary metastasectomy and all 5 patients were alive and well at the time of reporting. There was no significant difference in overall survival between patients having surgery or chemotherapy, but the numbers were small and the trend favoured surgery (81.7 % versus 49.5 %; *p* = 0.072). There was a significant survival advantage for patients with a disease-free survival of > 24 months (*p* =0.006).

## Surgical management of brain metastases

Brain metastases are common with breast, lung, and renal carcinomas, and malignant melanoma [30, 31], but are rare with gynaecological cancers, with the exception of choriocarcinoma [32, 33]. They are usually associated with widely disseminated disease.

### Diagnosis

Symptoms of brain metastases may be subtle initially, and may include headaches, nausea, vomiting, confusion, dizziness, decreased mental status, general or extremity weakness, urinary incontinence, gait disturbance, ataxia, visual disturbance including diplopia, photophobia, speech impairment, syncope or seizures [32–36]. Increased intracranial pressure caused by associated brain edema leads to the development of papilledema in the fundus of the eye, which is a classical sign of a brain tumor [37].

### Imaging

**Fig. 1:**
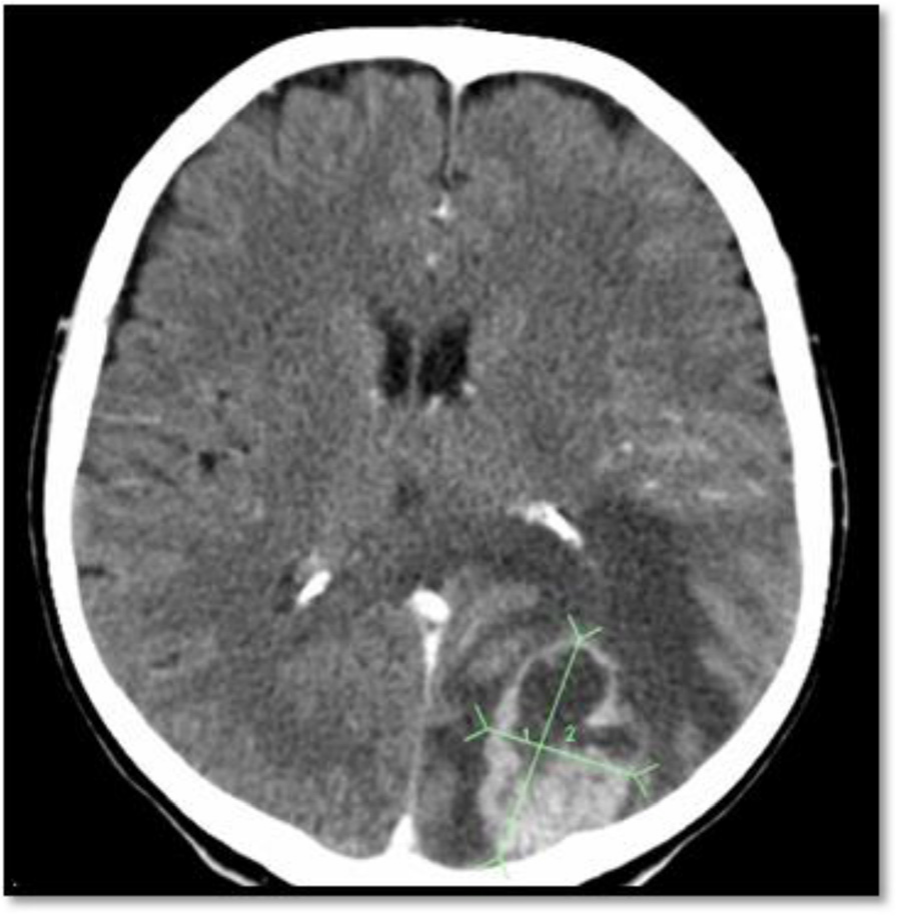
CT scan of the brain showing a solitary metastasis, 5x4 cm, in the right occipital lobe, with some extension to the parietal lobe. Note the heterogeneic appearance of the metastasis and the surrounding brain edema

Most brain metastases are diagnosed with a computed tomographic (CT) scan of the brain, which has been performed to investigate suspicious symptoms. The metastasis appears as a heterogeneous, contrast enhancing lesion [35] (Fig. 1). Metastatic ovarian cancers can occasionally be calcified [38]. Contrast-enhanced magnetic resonance imaging (MRI) is the most accurate modality to image the brain [39].

### Surgical Technique

Traditionally, patients with solitary metastases have undergone metastasectomy and whole brain radiotherapy (WBRT) [37, 39]. The latter is associated with a number of late complications, including brain atrophy, necrosis, dementia, and endocrine dysfunction [40].

More recently, stereotactic radiosurgery using a “gamma-knife” (GKRT) has become available [37, 41, 42]. This is a technique that enables the precise delivery of a high-dose of gamma radiation to a small intracranial target, while sparing the surrounding normal brain. If there is a solitary lesion, surgical resection followed by GKRT to the tumor bed is ideal. GKRT offers an advantage if the lesion is inaccessible. Lesions larger than 3 cm are less likely to be controlled by GKRT [41].

### Indications for surgical resection

Ideally, suitable patients for brain metastasectomy would have a solitary lesion and no evidence of extracranial disease [35, 43–45]. Such patients are uncommon, as the majority of patients have multiple brain metastases [39, 46–49]. A well controlled primary tumor [42, 50] and a disease-free interval of at least 12 months is desirable, but Petru et al. reported a patient who had a solitary brain metastasis diagnosed prior to the diagnosis of endometrial cancer. She had stereotactic radiosurgery to the brain lesion, followed by aggressive cytoreductive surgery and doxorubin-based chemotherapy for the primary tumor, and remained alive and free of disease at 171 months [51].

### Brain metastases by primary site

There have been comprehensive reviews by Piura and Piura regarding brain metastases from gynaecological cancers [32, 33, 37]. The incidence, pattern of brain metastasis, and survival by primary site are summarised in Tables 2 and 3.

**Table 2:** Summary of incidence, disease-free survival, and pattern of brain metastasis [32, 33, 37]

Primary Site	Incidence	Median Disease-Free Interval	Only site of metastatic disease	Solitary metastasis
Ovarian (*n* = 521)	1.19 % (413/34 728)	24.3 months (11–46)	46.8 % (236/504)	41.9 % (205/489)
Endometrial (*n* = 115)	0.59 % (61/10 199)	17 months (2–108)	49 % (48/98)	56.8 % (50/88)
Cervical (*n* = 100)	0.57 % (*n* = 65/11 249)	18 months (0.25-105 months)	46.8 % (37/79)	50.6 % (40/79)

**Table 3:** Summary of survival outcomes after diagnosis of brain metastases [32, 33, 37]

Primary Site	Median survival (months)	Surgery alone (months)	WBRT (months)	Surgery + WBRT (months)	Multimodal Surgery + RT +/− Chemo (months)
Ovarian	6.4 (1–28)	6.7	4.5	17	20
Endometrial	5 (0.1-171)	2.25 (1–18)	2 (0.25-17)	Not available	22 (2.1-84)
Cervical	4 (0.1-72)	4 (1–7)	3 (0.1-22.6) (+/− chemo)	7.1 (1–72) (+/− chemo)	SRS + other modality13.7 (5–22.5)

*WBRT* whole brain radiotherapy, *RT* radiotherapy, *SRS* stereotactic radiosurgery

#### Ovarian cancer

Of the gynaecological cancers, ovarian cancer is associated with the highest incidence of brain metastases, and there have been two comprehensive reviews in the past 5 years. In 2011, Piura and Piura reported 521 cases between 1978 and 2011 [32], while in 2014, Pakneshan et al. reported 591 cases between 1978 and 2013 [45]. Piura and Piura determined the incidence of brain metastases from ovarian cancer to be 1.2 % [32], which was twice the incidence associated with cervical [37] or endometrial cancer [33]. Pakneshan et al. reported that the incidence among the various studies ranged from 0.49 to 11.4 %, with an average of 2.55 % [45]. A review of the literature reveals several case reports and small series, the largest being 72 patients [47].

Since the advent of chemotherapy over 50 years ago, the incidence of brain metastases from ovarian cancer has increased, presumably because these patients are living longer, and because the chemotherapy has difficulty crossing the blood–brain barrier [31]. A review of 3,690 patients with epithelial ovarian cancer treated at the Royal Marsden Hospital from 1980 to 2000 reported that the incidence of brain metastases increased from 0.2 % in 1980–84 to 1.3 % in 1995–99 (*p* < 0.001) [34].

Most patients have advanced stage, high-grade serous cancers at initial presentation [35, 45, 47, 48, 52–54], and the brain metastasis often follows a negative second-look laparotomy [54–56]. CA125 titers are not absolutely reliable in the screening for brain metastases [57], although they are elevated in the majority of patients [45, 46].

In the review by Piura and Piura, the median interval from diagnosis to brain metastasis in 31 series was 24.3 months (range 11 to 46 months) [32], although there was a case report of a patient who developed a brain metastasis 11 years after diagnosis of the primary cancer [58]. The disease was confined to the central nervous system in 236 of 504 patients (46.8 %) [32]. The brain parenchyma, most commonly the cerebrum, was the site of metastasis in 489 patients (97 %) and the leptomeninges in 15 cases (3 %). Most brain metastases were multiple (269 of 474; 56.8 %) [32].

#### Prognosis

Survival by treatment type in patients with brain metastases from ovarian cancer is summarised in Table 4. Surgical resection significantly improved the survival compared to other methods of treatment [35, 43, 45, 47, 48, 52, 54, 59, 60]. Solitary metastases generally have a better prognosis [35, 43, 45]. Cormio et al. reported 22 patients who had resection of a solitary metastasis [44]. They reported that extracranial disease and the time interval between diagnosis of ovarian cancer and central nervous system involvement were the only factors significantly affecting survival. There were no operative deaths, and low morbidity. The majority of patients had complete resolution of their neurological symptoms [44].

**Table 4:** Ovarian cancer with brain metastases – survival by treatment modality after diagnosis of brain metastases [32]

Treatment modality	Median survival (months)	% of patients (n) Total = 538
WBRT^*^ only	4.5	35 % (182)
Surgery + WBRT	17	15.2 % (79)
WBRT + chemo	9.1	13.5 % (70)
Surgery + WBRT + chemo	20	13.3 % (69)
Surgery only	6.7	5 % (26)
SRS^*^ or GKRS^*^	18	3.8 % (20)
Chemo only	7.5	1.9 % (10)
Surgery + chemo	Not available	1.3 % (7)
No treatment (steroids only)	1.4	11 % (57)

*WBRT whole brain radiationtherapy, SRS stereotactic radiosurgery, GKRS gamma knife radiosurgery

Aggressive management of multiple metastases is justified [39, 46–49], particularly if the patient meets the criteria for Class I of the Radiation Therapy Oncology Group’s recursive partitioning analysis system, ie., age < 65 years, Karnofsky score > 70, controlled primary disease and no extracranial metastases [43]. Kawana et al. described a patient who was initially diagnosed with stage IVB ovarian cancer, and developed 3 brain metastases after a disease-free interval of 27 months [41]. She underwent surgical resection of the two accessible lesions, and then gamma-knife radiotherapy for a third inaccessible lesion after 30Gy external beam local radiation to the bed of the resected tumors and the inoperable tumor. She remained disease-free at 5 years, with good quality of life [41].

In their recent literature review, Piura and Piura reported that the median survival for patients having whole brain radiation was 4.5 months, compared to 17 months for patients having surgical resection plus radiation, and 20 months for the addition of chemotherapy [32]. The outcomes for patients with brain metastases by treatment modality are summarised in Table 4. In the review by Pakneshan et al., combination surgery, radiation and chemotherapy was associated with longer survival than whole brain radiation alone (20.5 months versus 9.1 months; *p* = 0.04) [45]. Others have also stressed the need for aggressive multimodal therapy, including adjuvant chemotherapy [47, 52, 59–62], as the patients usually succumb to extracranial disease [63].

Long term survival is possible. Micha el al reported a patient with a stage IIIC high-grade serous carcinoma who recurred in the cerebellum 27 months after diagnosis, and following primary cytoreductive surgery and platinum-based chemotherapy [64]. She had surgery and whole brain radiation and was still alive and well 7 years post treatment for her brain metastasis. McMeekin also described a 7-year survivor [60].

#### Cervical cancer

The most common site of distant metastases from cervical cancer is the lung [65, 66]. Brain metastases are rare. In a literature review in 2012, Piura and Piura reported only 96 cases, with an incidence of 0.57 % [37]. The majority of patients had early stage disease at diagnosis – 42.2 % had stage IB and 36.6 % stage II – although 80 % of patients had poorly differentiated (grade 3) tumors. Histologic types basically reflected those expected in the general population, although there were 3 (3.6 %) small cell neuroendocrine carcinomas [37].

The interval between primary diagnosis and brain metastasis ranged from 1 week to 105 months, with a median of 18 months. The brain metastasis was part of a disseminated recurrence in 53.2 % of patients, was solitary in 50.6 %, and was located only in the cerebrum in 73 % of patients. Some authors have noted that brain metastases from cervical cancer were rarely accompanied by systemic disease, but they were commonly accompanied by uncontrolled local-regional disease [42, 50].

#### Prognosis

Based on limited data in the literature, Piura and Piura determined that the median survival for no treatment was 0.6 months, for whole brain radiation (WBRT) 4 months, while for surgical resection followed by WBRT it was 7.1 months. The best median survival (13.7 months) was achieved with stereotactic radiation, either alone or combined with another modality [37].

Robinson and Morris reported a patient with a brain metastasis from a squamous cell carcinoma of the cervix who remained disease free 6 years following surgical resection and whole brain radiation [67]. Chura et al. reported 12 patients with brain metastases from cervical cancer, 8 of whom received WBRT, but their median survival was only 2.3 months [68].

#### Endometrial Cancer

Brain metastases from endometrial cancer are rare, with Piura and Piura documenting only 115 cases from 35 published papers, with an incidence of 0.59 % [33]. The brain metastasis was diagnosed after a median interval of 17 months (range 2 to 108 months). In 4 patients (4.2 %), the primary and metastatic diagnoses were made simultaneously, and in 9 patients (9.5 %), the brain metastases were detected before the primary.

In the review by Piura and Piura, 63 % of patients (50 of 79) had advanced disease at initial diagnosis, and 78.1 % (57 of 73) had poorly differentiated tumors [33]. Almost half the patients had metastases confined to the brain (48 of 98 patients; 49 %), and 56.8 % of patients (50 of 88) had a solitary metastasis. Site of metastasis was available for 66 patients, of whom 48 (72.7 %) had disease confined to the cerebellum [33]. Of the 20 patients reported by Chura et al., 8 (40 %) had a single metastasis, 4 (20 %) had two, 7 (35 %) had 3 or more, and 1 patient (5 %) had leptomeningeal disease [69].

#### Prognosis

In the review by Piura and Piura, the overall median survival after diagnosis of brain metastasis was 5 months (0.1 to 171 months). Patients having WBRT alone had a median survival of 2 months (0.25-17 months) while patients having surgical resection followed by WBRT had a median survival of 22 months (2.1-84 months) [33]. Orrru et al. reported 2 patients treated by surgical resection followed by WBRT who were alive and well at 16 and 64 months respectively [70].

## Surgical management of liver metastases

The literature on liver resection for metastatic gynecological cancer is limited. It has been estimated that up to 50 % of patients who die of cervical, endometrial or ovarian cancer will have liver metastases at autopsy [71, 72], but probably only 1 - 10 % would be suitable for liver resection [73]. Gynecologic cancers that metastasize to the liver usually do so in the setting of obvious regional or systemic dissemination [71, 74].

### Surgical technique

Hepatic resection has evolved, with improved surgical techniques, instrumentation, anesthesia and perioperative care, and now carries a very low morbidity and mortality [73, 75, 76].

**Fig. 2:**
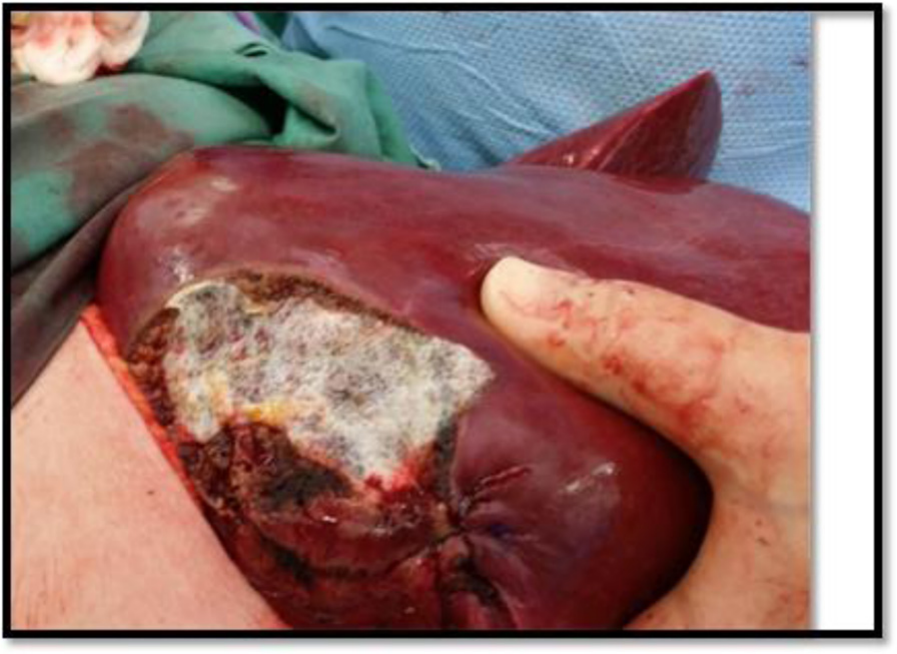
Non-anatomical liver resection for a patient with ovarian cancer with involvement of the liver capsule and underlying parenchyma

Hepatic resection usually involves non-anatomical wedge resection (Fig. 2), but anatomical resection of one or more liver segments may also be performed [77]. Resection of as much as 70 % of the liver can be performed, with a mortality rate of less than 5 % in major hepatobiliary centers [77, 78]. Over the past 15 years, radiofrequency ablation, usually in conjunction with surgical resection, has extended the cohort of patients with surgically treatable disease, and helped achieve better locoregional control [79, 80].

### Indications

Liver surgery should only be considered when all other metastatic disease is well controlled, when disease in the liver can be completely resected, or when liver resection is part of the achievement of optimal cytoreduction for patients with ovarian cancer [81, 82].

## Results

The findings of reported series with 5 or more patients are summarised in Table 5.

**Table 5:** Major series reporting surgical management of hepatic metastases from gynaecological malignancies

First Author Year of Publication	Number of patients	Primary vs Recurrent Disease	Primary Site	Median overall survival (from time of liver resection unless otherwise stated)	Factors associated with longest survival
Kolev [91] 2014	27	Recurrent	Ovary	12 months (2–190)	Interval from primary surgery of >24 months (*P* = 0.044) Secondary cytoreduction to <1 cm (*P* = 0.014)
Neumann [82] 2012	41	Primary	Ovary	R0 – 42 months R1 – 4 months R2 – 6 months	Post operative residual tumour mass
Roh [77] 2011	18	Recurrent	Ovary	38 months (3–78)	Less abdominal than pelvic disease (38 vs 11 months, *P* = 0.032) Optimal cytoreduction (40 vs 9 months, *P* = 0.0004) Negative margin status of hepatic resection (40 vs (months, *P* = 0.0196
Kamel [92] 2011	52	Primary	Ovary	53 months 5-year survival 41 %	Not reported
Knowles [93] 2010	5	Recurrent	Endometrioid (Ovarian or Endometrial)	Median OS not reported DFS range 8–66 months	Not reported
Lim [75] 2009	14	Primary	Ovary	5-year PFS by Stage: - IIIC – 25 % - IV – 23 % 5-year OS by Stage: - IIIC – 55 % - IV – 51 %	Not reported
Loizzi [94] 2005	29	Primary (Group 1) – 8 1^st^ Recurrence (Group 2) – 10 2^nd^ recurrence (Group 3) – 11	Ovary	Median survival from time of liver metastasis diagnosis: Group 1 – 19 months Group 2 – 24 months Group 3 – 10 months	Cell type Performance status Number of hepatic lesions Presence of other sites of disease at time of diagnosis of hepatic metastasis Platinum based chemotherapy
Weitz [76] 2005	19	Recurrent	Ovary – 63.2 % Endometrium – 21.1 % Cervix – 10.5 % Fallopian tube – 5.2 %	Reproductive tract tumours (note – included testicular cancer pts, but no difference between ovary and testicular survival) Median cancer specific survival reproductive tract primary – 115 months Ovary - 3 year recurrence free survival 58 %	Primary tumour type Length of disease free interval from primary tumour
Yoon [95] 2003	24	Recurrent	Ovary Fallopian tube	62 months (6–94)	No significant prognostic factors for OS identified on univariate analysis
Merideth [89] 2003	26	Recurrent	Ovary	Overall median disease-related survival 26.3 months	>12 months since original diagnosis (27.3 vs 5.7 months, *P* = 0.004) ≤1 cm residual disease (27.3 vs 8.6 months, *P* = 0.031)
Fan [96] 2001	18		Ovary – immature teratoma	3-year survival – 77.8 % 5-year survival – 55.6 % 10-year survival – 38.9 %	Not reported
Naik [97] 2000	37	Primary	Ovary	11 months 2-year survival 23 % 5-year survival 9 %	Optimal surgery with residual <2 cm (*P* = 0.0029) or <1 cm (*P* = 0.0086)
Bristow [81] 1999	37	Primary	Ovary	Optimal extrahepatic and hepatic resection – 50.1 months Optimal extrahepatic resection with residual hepatic tumour – 27.0 months Suboptimal with residual extrahepatic and heaptic tumour – 7.6 months	Optimal extrahepatic resection (*P* = 0.0001)
Elias [84] 1998	6	Not stated	Gynecologic	5-year survival – 45 %	Not reported
Chi [71] 1997	12	Recurrent	Ovary – 58 % Cervix – 17 % Endometrium – 17 % Fallopian tube – 1 8 %	27 months (Median f/up 25 months, range 8–94 months)	Not reported

*OS* overall survival, *DFS* disease-free survival, *PFS* progression-free survival

The first study by Brunchwig in 1963 [83] reported 24 cases of hepatic lobectomy for metastatic carcinoma, 4 of whom were from the cervix or endometrium. Three of the four died in the perioperative period, and the fourth died of disease at 18 months..

In most large series of patients having partial hepatectomy for metastatic malignancy, gynaecological cancers represent less than 10 % of cases [73, 76, 84–86]. A large, multi-centre French study reported that during the 1980’s, the median number of partial hepatectomies for non-colorectal, non-endocrine metastases per annum did not exceed 17, whereas it rose to 70 during the 1990’s and 115 during the 2000’s [73].

In patients with colorectal and gut-associated endocrine tumors, the most likely mode of spread is via the portal venous system. The majority of the patient’s tumor burden is thus confined to the abdomen [73], and 5-year survivals of 45-50 % are routinely reported [87, 88]. By contrast, liver metastases from gynaecological cancers reach the liver via the systemic circulation, so other extra-abdominal sites are likely to be involved, which has fostered caution. Liver surgery should only be considered when the metastatic disease is well controlled or responding to systemic therapy, or when liver resection is part of the achievement of optimal cytoreduction for ovarian cancer [81, 82].

Lim et al. reported 16 patients who had parenchymal liver metastases at the time of diagnosis with advanced ovarian cancer [75]. Two patients (12.5 %) had hematogenous metastases which were unresectable, while 14 (87.5 %) had parenchymal invasion from peritoneal seeding, and were able to undergo complete resection. These patients, who were officially FIGO stage IV, had the same survival as patients with Stage IIIC disease.

A series of 26 patients undergoing hepatic resection for metachronous metastases from ovarian cancer was reported from the Mayo Clinic in 2003 [89]. The median age of the patients was 62 years and a solitary liver lesion was present in 17 patients (63.4 %). All patients had pelvic and abdominal disease in addition to the liver metastases and 42 % had a simultaneous bowel resection. Optimal cytoreduction (<1 cm) was achieved in 21 patients (80.7 %). Segmentectomy was required in 18 patients (69.2 %) and right hepatectomy in 4 (15.4 %). There was no serious morbidity or mortality from the surgery. There was a significant survival advantage for patients whose disease-free interval was >12 months (27.3 versus 5.7 months; *p* < 0.004)), and for those having optimal cytoreduction (27.3 versus 8.6 months; *p* < 0.031)

In 2006, a multicentre French study reported the largest series (1452) of patients who underwent hepatic resection for non-colorectal, non-endocrine liver metastases [73]. Gynecologic cancer represented 126 cases (8.7 %), and although the patients were highly selected, the results were very satisfactory. Overall, the 5-year survival was 48 %. It was 50 % for patients with ovarian cancer and 35 % for patients with a uterine primary [73]. For the 1452 patients the 60-day operative mortality was 2.3 %.

## Conclusions

From a review of the current literature, it is apparent that surgery should play an increasing role in the management of patients with parenchymal metastases from gynecological cancers to the lungs, brain or liver. Appropriate patient selection is critical, but surprisingly good long-term survival results can be achieved for resection of metastases from all three organs, in conjunction usually with the use of adjuvant radiation, chemotherapy or hormonal therapy.

This requires the development of a committed, multidisciplinary team, working in a high volume tertiary center, where the necessary infrastructure for postoperative management is available. In these circumstances, postoperative morbidity and mortality are low.

Ideally, patients should have metastatic disease confined to the lungs, brain or liver, except in the case of a patient with a chemosensitive ovarian cancer, where resection of pulmonary or liver metastases may form part of the initial cytoreductive surgical effort. A solitary metastasis is ideal, but good results may be obtained with multiple metastases, as long as all macroscopic disease can be resected. A disease-free interval of at least 12 months, and preferably 24 months is desirable, together with a satisfactory performance status, and adequate functional reserve in the organ being partially resected.

## References

[1] Barney JD, Churchill CE (1939). Adenocarcinoma of the kidney with metastasis to the lung. J Urol.

[2] Mountain CF, McMurtrey MJ, Hermes KE (1984). Surgery for pulmonary metastasis: a 20-year experience. Ann Thorac Surg.

[3] Casson AG, Putnam JB, Natarajan G, Johnston DA, Mountain C, McMurtrey M, Roth JA (1992). Five-year survival after pulmonary metastasectomy for adult soft tissue sarcoma. Cancer.

[4] Headrick JR, Miller DL, Nagorney DM, Allen MS, Deschamps C, Trastek VF, Pairolero PC (2001). Surgical treatment of hepatic and pulmonary metastases from colon cancer. Ann Thorac Surg.

[5] Martini N, McCormack PM (1998). Evolution of the surgical management of pulmonary metastases. Chest Surg Clin N Am.

[6] Monteiro A, Arce N, Bernardo J, Eugenio L, Antunes MJ (2004). Surgical resection of lung metastases from epithelial tumors. Ann Thorac Surg.

[7] Murthy SC, Kim K, Rice TW, Rajeswaran J, Bukowski R, DeCamp MM, Blackstone EH (2005). Can we predict long-term survival after pulmonary metastasectomy for renal cell carcinoma?. Ann Thorac Surg.

[8] Piltz S, Meimarakis G, Wichmann MW, Hatz R, Schildberg FW, Fuerst H (2002). Long-term results after pulmonary resection of renal cell carcinoma metastases. Ann Thorac Surg.

[9] Pastorino U, Buyse M, Friedel G, Ginsberg RJ, Girard P, Goldstraw P, Johnston M, McCormack P, Pass H, Putnam JB, International Registry of Lung M (1997). Long-term results of lung metastasectomy: prognostic analyses based on 5206 cases. J Thoracic & Cardiovasc Surg.

[10] Creasman WT, Odicino F, Maisonneuve P, Quinn MA, Beller U, Benedet JL, Heintz AP, Ngan HY, Pecorelli S (2006). Carcinoma of the corpus uteri. FIGO 26th Annual Report on the Results of Treatment in Gynecological Cancer. Int J Gynaecology & Obstetrics.

[11] Blecharz P, Urbanski K, Mucha-Malecka A, Malecki K, Reinfuss M, Jakubowicz J, Skotnicki P (2011). Hematogenous metastases in patients with Stage I or II endometrial carcinoma. Strahlenther Onkol.

[12] Bouros D, Papadakis K, Siafakas N, Fuller AF (1996). Patterns of pulmonary metastasis from uterine cancer. Oncology.

[13] Labi FL, Evangelista S, Di Miscia A, Stentella P (2008). FIGO Stage I endometrial carcinoma: evaluation of lung metastases and follow-up. Eur J Gynaecol Oncol.

[14] Torre LA, Bray F, Siegel RL, Ferlay J, Lortet-Tieulent J, Jemal A (2015). Global cancer statistics, 2012. CA Cancer J Clin.

[15] Gunasekera PC (1999). Emergency contraception. Ceylon Med J.

[16] Barter JF, Soong SJ, Hatch KD, Orr JW, Shingleton HM (1990). Diagnosis and treatment of pulmonary metastases from cervical carcinoma. Gynecol Oncol.

[17] Martinez-Jimenez S, Rosado-de-Christenson ML, Walker CM, Kunin JR, Betancourt SL, Shoup BL, Pettavel PP (2014). Imaging features of thoracic metastases from gynecologic neoplasms. Radiographics.

[18] Paramanathan A, Wright G (2013). Pulmonary metastasectomy for sarcoma of gynaecologic origin. Heart Lung Circ.

[19] Gonzalez Casaurran G, Simon Adiego C, Penalver Pascual R, Moreno Mata N, Lozano Barriuso MA, Gonzalez Aragoneses F (2011). Surgery of female genital tract tumour lung metastases. Arch Bronconeumol.

[20] Anderson TM, McMahon JJ, Nwogu CE, Pombo MW, Urschel JD, Driscoll DL, Lele SB (2001). Pulmonary resection in metastatic uterine and cervical malignancies. Gynecol Oncol.

[21] Anraku M, Yokoi K, Nakagawa K, Fujisawa T, Nakajima J, Akiyama H, Nishimura Y, Kobayashi K, Metastatic Lung Tumor Study Group of J (2004). Pulmonary metastases from uterine malignancies: results of surgical resection in 133 patients. J Thoracic & Cardiovasc Surg.

[22] Burt BM, Ocejo S, Mery CM, Dasilva M, Bueno R, Sugarbaker DJ, Jaklitsch MT (2011). Repeated and aggressive pulmonary resections for leiomyosarcoma metastases extends survival. Ann Thorac Surg.

[23] Clavero JM, Deschamps C, Cassivi SD, Allen MS, Nichols FC, Barrette BA, Larson DR, Pairolero PC (2006). Gynecologic cancers: factors affecting survival after pulmonary metastasectomy. Ann Thorac Surg.

[24] Seki M, Nakagawa K, Tsuchiya S, Matsubara T, Kinoshita I, Weng SY, Tsuchiya E (1992). Surgical treatment of pulmonary metastases from uterine cervical cancer. Operation method by lung tumor size. J Thorac Cardiovasc Surg.

[25] Fuller AF, Scannell JG, Wilkins EW (1985). Pulmonary resection for metastases from gynecologic cancers: Massachusetts General Hospital experience, 1943–1982. Gynecol Oncol.

[26] McCormack PM, Martini N (1979). The changing role of surgery for pulmonary metastases. Ann Thorac Surg.

[27] Levenback C, Rubin SC, McCormack PM, Hoskins WJ, Atkinson EN, Lewis JL (1992). Resection of pulmonary metastases from uterine sarcomas. Gynecol Oncol.

[28] Yamamoto K, Yoshikawa H, Shiromizu K, Saito T, Kuzuya K, Tsunematsu R, Kamura T (2004). Pulmonary metastasectomy for uterine cervical cancer: a multivariate analysis. Ann Thorac Surg.

[29] Adachi M, Mizuno M, Mitsui H, Kajiyama H, Suzuki S, Sekiya R, Utsumi F, Shibata K, Taniguchi T, Kawaguchi K (2015). The prognostic impact of pulmonary metastasectomy in recurrent gynecologic cancers: a retrospective single-institution study. Nagoya J Med Sci.

[30] Schouten LJ, Rutten J, Huveneers HA, Twijnstra A (2002). Incidence of brain metastases in a cohort of patients with carcinoma of the breast, colon, kidney, and lung and melanoma. Cancer.

[31] Tosoni A, Ermani M, Brandes AA (2004). The pathogenesis and treatment of brain metastases: a comprehensive review. Crit Rev Oncol Hematol.

[32] Piura E, Piura B (2011). Brain metastases from ovarian carcinoma. ISRN Oncol.

[33] Piura E, Piura B (2012). Brain metastases from endometrial carcinoma. ISRN Oncol.

[34] Kolomainen DF, Larkin JM, Badran M, A'Hern RP, King DM, Fisher C, Bridges JE, Blake PR, Barton DP, Shepherd JH (2002). Epithelial ovarian cancer metastasizing to the brain: a late manifestation of the disease with an increasing incidence. J Clin Oncol.

[35] LeRoux PD, Berger MS, Elliott JP, Tamimi HK (1991). Cerebral metastases from ovarian carcinoma. Cancer.

[36] Plaxe SC, Dottino PR, Lipsztein R, Dalton J, Cohen CJ (1990). Clinical features and treatment outcome of patients with epithelial carcinoma of the ovary metastatic to the central nervous system. Obstet Gynecol.

[37] Piura E, Piura B (2012). Brain metastases from cervical carcinoma: overview of pertinent literature. Eur J Gynaecol Oncol.

[38] Kawamura D, Tanaka T, Fuga M, Yanagisawa T, Tochigi S, Irie K, Hasegawa Y, Abe T (2013). Slow progression of calcified cerebellar metastasis from ovarian cancer: a case report and review of the literature. Neurol Med Chir.

[39] Kim TJ, Song S, Kim CK, Kim WY, Choi CH, Lee JH, Lee JW, Bae DS, Kim BG (2007). Prognostic factors associated with brain metastases from epithelial ovarian carcinoma. Int J Gynecol Cancer.

[40] Schultheiss TE, Kun LE, Ang KK, Stephens LC (1995). Radiation response of the central nervous system. Int J Radiat Oncol Biol Phys.

[41] Kawana K, Yoshikawa H, Yokota H, Onda T, Nakagawa K, Tsutsumi O, Taketani Y (1997). Successful treatment of brain metastases from ovarian cancer using gamma-knife radiosurgery. Gynecol Oncol.

[42] Mahmoud-Ahmed AS, Suh JH, Barnett GH, Webster KD, Kennedy AW (2001). Tumor distribution and survival in six patients with brain metastases from cervical carcinoma. Gynecol Oncol.

[43] Chen PG, Lee SY, Barnett GH, Vogelbaum MA, Saxton JP, Fleming PA, Suh JH (2005). Use of the Radiation Therapy Oncology Group recursive partitioning analysis classification system and predictors of survival in 19 women with brain metastases from ovarian carcinoma. Cancer.

[44] Cormio G, Maneo A, Colamaria A, Loverro G, Lissoni A, Selvaggi L (2003). Surgical resection of solitary brain metastasis from ovarian carcinoma: an analysis of 22 cases. Gynecol Oncol.

[45] Pakneshan S, Safarpour D, Tavassoli F, Jabbari B (2014). Brain metastasis from ovarian cancer: a systematic review. J Neurooncol.

[46] Anupol N, Ghamande S, Odunsi K, Driscoll D, Lele S (2002). Evaluation of prognostic factors and treatment modalities in ovarian cancer patients with brain metastases. Gynecol Oncol.

[47] Cohen ZR, Suki D, Weinberg JS, Marmor E, Lang FF, Gershenson DM, Sawaya R (2004). Brain metastases in patients with ovarian carcinoma: prognostic factors and outcome. J Neurooncol.

[48] Geisler JP, Geisler HE (1995). Brain metastases in epithelial ovarian carcinoma. Gynecol Oncol.

[49] Ratner ES, Toy E, O'Malley DM, McAlpine J, Rutherford TJ, Azodi M, Higgins SA, Schwartz PE (2009). Brain metastases in epithelial ovarian and primary peritoneal carcinoma. Int J Gynecol Cancer.

[50] Cormio G, Colamaria A, Loverro G, Pierangeli E, Di Vagno G, De Tommasi A, Selvaggi L (1999). Surgical resection of a cerebral metastasis from cervical cancer: case report and review of the literature. Tumori.

[51] Petru E, Lax S, Kurschel S, Gucer F, Sutter B (2001). Long-term survival in a patient with brain metastases preceding the diagnosis of endometrial cancer. Report of two cases and review of the literature. J Neurosurg.

[52] Kaminsky-Forrett MC, Weber B, Conroy T, Spaeth D (2000). Brain metastases from epithelial ovarian carcinoma. Int J Gynecol Cancer.

[53] Pectasides D, Pectasides M, Economopoulos T (2006). Brain metastases from epithelial ovarian cancer: a review of the literature. Oncologist.

[54] Pothuri B, Chi DS, Reid T, Aghajanian C, Venkatraman E, Alektiar K, Bilsky M, Barakat RR (2002). Craniotomy for central nervous system metastases in epithelial ovarian carcinoma. Gynecol Oncol.

[55] Akhan SE, Isikoglu M, Salihoglu Y, Bengisu E, Berkman S (2002). Brain metastasis of ovarian cancer after negative second-look laparotomy. Eur J Gynaecol Oncol.

[56] Deutsch M, Beck D, Manor D, Brandes J (1987). Metastatic brain tumor following negative second-look operation for ovarian carcinoma. Gynecol Oncol.

[57] Tay SK, Rajesh H (2005). Brain metastases from epithelial ovarian cancer. Int J Gynecol Cancer.

[58] Longo R, Platini C, Eid N, Elias-Matta C, Buda T, Nguyen D, Quetin P (2014). A late, solitary brain metastasis of epithelial ovarian carcinoma. BMC Cancer.

[59] Cormio G, Loizzi V, Falagario M, Lissoni AA, Resta L, Selvaggi LE (2011). Changes in the management and outcome of central nervous system involvement from ovarian cancer since 1994. International Journal of Gynaecology & Obstetrics.

[60] McMeekin DS, Kamelle SA, Vasilev SA, Tillmanns TD, Gould NS, Scribner DR, Gold MA, Guruswamy S, Mannel RS (2001). Ovarian cancer metastatic to the brain: what is the optimal management?. J Surg Oncol.

[61] D'Andrea G, Roperto R, Dinia L, Caroli E, Salvati M, Ferrante L (2005). Solitary cerebral metastases from ovarian epithelial carcinoma: 11 cases. Neurosurg Rev.

[62] Rodriguez GC, Soper JT, Berchuck A, Oleson J, Dodge R, Montana G, Clarke-Pearson DL (1992). Improved palliation of cerebral metastases in epithelial ovarian cancer using a combined modality approach including radiation therapy, chemotherapy, and surgery. J Clin Oncol.

[63] Kastritis E, Efstathiou E, Gika D, Bozas G, Koutsoukou V, Papadimitriou C, Pissakas G, Dimopoulos MA, Bamias A (2006). Brain metastases as isolated site of relapse in patients with epithelial ovarian cancer previously treated with platinum and paclitaxel-based chemotherapy. Int J Gynecol Cancer.

[64] Micha JP, Goldstein BH, Hunter JV, Rettenmaier MA, Brown JV (2004). Long-term survival in an ovarian cancer patient with brain metastases. Gynecol Oncol.

[65] Bodurka-Bevers D, Morris M, Eifel PJ, Levenback C, Bevers MW, Lucas KR, Wharton JT (2000). Posttherapy surveillance of women with cervical cancer: an outcomes analysis. Gynecol Oncol.

[66] Elit L, Fyles AW, Devries MC, Oliver TK, Fung-Kee-Fung M, Gynecology Cancer Disease Site G (2009). Follow-up for women after treatment for cervical cancer: a systematic review. Gynecol Oncol.

[67] Robinson JB, Morris M (1997). Cervical carcinoma metastatic to the brain. Gynecol Oncol.

[68] Chura JC, Shukla K, Argenta PA (2007). Brain metastasis from cervical carcinoma. Int J Gynecol Cancer.

[69] Chura JC, Marushin R, Boyd A, Ghebre R, Geller MA, Argenta PA (2007). Multimodal therapy improves survival in patients with CNS metastasis from uterine cancer: a retrospective analysis and literature review. Gynecol Oncol.

[70] Orrru S, Lay G, Dessi M, Murtas R, Deidda MA, Amichetti M (2007). Brain metastases from endometrial carcinoma: report of three cases and review of the literature. Tumori.

[71] Chi DS, Fong Y, Venkatraman ES, Barakat RR (1997). Hepatic resection for metastatic gynecologic carcinomas. Gynecol Oncol.

[72] Rose PG, Piver MS, Tsukada Y, Lau TS (1989). Metastatic patterns in histologic variants of ovarian cancer. An autopsy study. Cancer.

[73] Adam R, Chiche L, Aloia T, Elias D, Salmon R, Rivoire M, Jaeck D, Saric J, Le Treut YP, Belghiti J (2006). Hepatic resection for noncolorectal nonendocrine liver metastases: analysis of 1,452 patients and development of a prognostic model. Ann Surg.

[74] Kim GE, Lee SW, Suh CO, Park TK, Kim JW, Park JT, Shim JU (1998). Hepatic metastases from carcinoma of the uterine cervix. Gynecol Oncol.

[75] Lim MC, Kang S, Lee KS, Han SS, Park SJ, Seo SS, Park SY (2009). The clinical significance of hepatic parenchymal metastasis in patients with primary epithelial ovarian cancer. Gynecol Oncol.

[76] Weitz J, Blumgart LH, Fong Y, Jarnagin WR, D'Angelica M, Harrison LE, DeMatteo RP (2005). Partial hepatectomy for metastases from noncolorectal, nonneuroendocrine carcinoma. Ann Surg.

[77] Roh HJ, Kim DY, Joo WD, Yoo HJ, Kim JH, Kim YM, Kim YT, Nam JH (2011). Hepatic resection as part of secondary cytoreductive surgery for recurrent ovarian cancer involving the liver. Archives of Gynecology & Obstetrics.

[78] Chang YC (2004). Low mortality major hepatectomy. Hepatogastroenterology.

[79] Goering JD, Mahvi DM, Niederhuber JE, Chicks D, Rikkers LF (2002). Cryoablation and liver resection for noncolorectal liver metastases. Am J Surg.

[80] Mateo R, Singh G, Jabbour N, Palmer S, Genyk Y, Roman L (2005). Optimal cytoreduction after combined resection and radiofrequency ablation of hepatic metastases from recurrent malignant ovarian tumors. Gynecol Oncol.

[81] Bristow RE, Montz FJ, Lagasse LD, Leuchter RS, Karlan BY (1999). Survival impact of surgical cytoreduction in stage IV epithelial ovarian cancer. Gynecol Oncol.

[82] Neumann UP, Fotopoulou C, Schmeding M, Thelen A, Papanikolaou G, Braicu EI, Neuhaus P, Sehouli J (2012). Clinical outcome of patients with advanced ovarian cancer after resection of liver metastases. Anticancer Res.

[83] Brunschwig A (1963). Hepatic lobectomy for metastatic cancer. Cancer.

[84] Elias D, Cavalcanti de Albuquerque A, Eggenspieler P, Plaud B, Ducreux M, Spielmann M, Theodore C, Bonvalot S, Lasser P (1998). Resection of liver metastases from a noncolorectal primary: indications and results based on 147 monocentric patients. J Am Coll Surg.

[85] Ercolani G, Grazi GL, Ravaioli M, Ramacciato G, Cescon M, Varotti G, Del Gaudio M, Vetrone G, Pinna AD (2005). The role of liver resections for noncolorectal, nonneuroendocrine metastases: experience with 142 observed cases. Ann Surg Oncol.

[86] O'Rourke TR, Tekkis P, Yeung S, Fawcett J, Lynch S, Strong R, Wall D, John TG, Welsh F, Rees M (2008). Long-term results of liver resection for non-colorectal, non-neuroendocrine metastases. Ann Surg Oncol.

[87] Adam R, Pascal G, Azoulay D, Tanaka K, Castaing D, Bismuth H (2003). Liver resection for colorectal metastases: the third hepatectomy. Ann Surg.

[88] Jaeck D, Bachellier P, Guiguet M, Boudjema K, Vaillant JC, Balladur P, Nordlinger B (1997). Long-term survival following resection of colorectal hepatic metastases. Association Francaise de Chirurgie. Br J Surg.

[89] Merideth MA, Cliby WA, Keeney GL, Lesnick TG, Nagorney DM, Podratz KC (2003). Hepatic resection for metachronous metastases from ovarian carcinoma. Gynecol Oncol.

[90] Lim MC, Lee HS, Seo SS, Kim MS, Kim JY, Zo JI, Park SY (2010). Pathologic diagnosis and resection of suspicious thoracic metastases in patients with cervical cancer through thoracotomy or video-assisted thoracic surgery. Gynecol Oncol.

[91] Kolev V, Pereira EB, Schwartz M, Sarpel U, Roayaie S, Labow D, Momeni M, Chuang L, Dottino P, Rahaman J, Zakashansky K (2014). The role of liver resection at the time of secondary cytoreduction in patients with recurrent ovarian cancer. Int J Gynecol Cancer.

[92] Kamel SI, de Jong MC, Schulick RD, Diaz-Montes TP, Wolfgang CL, Hirose K, Edil BH, Choti MA, Anders RA, Pawlik TM (2011). The role of liver-directed surgery in patients with hepatic metastasis from a gynecologic primary carcinoma. World J Surg.

[93] Knowles B, Bellamy CO, Oniscu A, Wigmore SJ (2010). Hepatic resection for metastatic endometrioid carcinoma. HPB.

[94] Loizzi V, Rossi C, Cormio G, Cazzolla A, Altomare D, Selvaggi L (2005). Clinical features of hepatic metastasis in patients with ovarian cancer. Int J Gynecol Cancer.

[95] Yoon SS, Jarnagin WR, Fong Y, DeMatteo RP, Barakat RR, Blumgart LH, Chi DS (2003). Resection of recurrent ovarian or fallopian tube carcinoma involving the liver. Gynecol Oncol.

[96] Fan Q, Huang H, Lian L, Lang J (2001). Characteristics, diagnosis and treatment of hepatic metastasis of pure immature ovarian teratoma. Chin Med J (Engl).

[97] Naik R, Nordin A, Cross PA, Hemming D, de Barros LA, Monaghan JM (2000). Optimal cytoreductive surgery is an independent prognostic indicator in stage IV epithelial ovarian cancer with hepatic metastases. Gynecol Oncol.

